# Coral Carbonic Anhydrases: Regulation by Ocean Acidification

**DOI:** 10.3390/md14060109

**Published:** 2016-06-03

**Authors:** Didier Zoccola, Alessio Innocenti, Anthony Bertucci, Eric Tambutté, Claudiu T. Supuran, Sylvie Tambutté

**Affiliations:** 1 Marine Biology Department, Centre Scientifique de Monaco, 8 Quai Antoine 1°, 98 000 Monaco, Monaco; zoccola@centrescientifique.mc (D.Z.); anthony.bertucci@u-bordeaux.fr (A.B.); etambutté@centrescientifique.mc (E.T.); 2 Laboratoire International Associé 647 BIOSENSIB, Centre Scientifique de Monaco-Centre National de la Recherche Scientifique, 8 Quai Antoine 1°, 98 000 Monaco, Monaco; 3 Neurofarba Department, University of Florence, Via Ugo Schiff 6, Polo Scientifico, Sesto Fiorentino, 50019 Firenze, Italy; alessio.innocenti@unifi.it

**Keywords:** coral, calcification, ocean acidification, carbonic anhydrase, gene expression, enzyme activity, temperature, pH

## Abstract

Global change is a major threat to the oceans, as it implies temperature increase and acidification. Ocean acidification (OA) involving decreasing pH and changes in seawater carbonate chemistry challenges the capacity of corals to form their skeletons. Despite the large number of studies that have investigated how rates of calcification respond to ocean acidification scenarios, comparatively few studies tackle how ocean acidification impacts the physiological mechanisms that drive calcification itself. The aim of our paper was to determine how the carbonic anhydrases, which play a major role in calcification, are potentially regulated by ocean acidification. For this we measured the effect of pH on enzyme activity of two carbonic anhydrase isoforms that have been previously characterized in the scleractinian coral *Stylophora pistillata*. In addition we looked at gene expression of these enzymes *in vivo.* For both isoforms, our results show (1) a change in gene expression under OA (2) an effect of OA and temperature on carbonic anhydrase activity. We suggest that temperature increase could counterbalance the effect of OA on enzyme activity. Finally we point out that caution must, thus, be taken when interpreting transcriptomic data on carbonic anhydrases in ocean acidification and temperature stress experiments, as the effect of these stressors on the physiological function of CA will depend both on gene expression and enzyme activity.

## 1. Introduction

Anthropogenic greenhouse gas emissions have increased since the pre-industrial era, which has led to an increase in atmospheric concentrations of carbon dioxide (CO_2_), methane, and nitrous oxide. Their effects are extremely likely to have been the dominant cause of the observed warming since the mid-20th century (IPCC, 2014) [1]. In addition to atmospheric and oceanic warming, the subsequent uptake of additional CO_2_ by the oceans causes ocean acidification (OA), which results in pH decrease and changes in seawater carbonate chemistry. Earth system models project a global increase in ocean acidification for all representative concentration pathway (RCP) scenarios by the end of the 21st century, with a slow recovery after mid-century under RCP2.6 (IPCC, 2014) [1]. The decrease in surface ocean pH is in the range of 0.06 to 0.07 (15% to 17% increase in acidity) for RCP2.6, 0.14 to 0.15 (38% to 41%) for RCP4.5, 0.20 to 0.21 (58% to 62%) for RCP6.0, and 0.30 to 0.32 (100% to 109%) for RCP8.5 (IPCC, 2014) [1]. Ocean acidification by decreasing pH and changing carbonate chemistry challenges marine organisms, especially those that form calcareous shells and skeletons, such as scleractinian corals, the major contributors to the structural foundation of coral-reef ecosystems. Meta-analysis of data obtained from laboratory and field-based studies indicate declines in coral calcification of 15%–22% at levels of OA predicted to occur under a business-as-usual scenario of CO_2_ emissions by the end of the century [2] (note that this scenario predicts a pCO_2_ of 800 ppm by the end of the century which corresponds to the prediction of scenario RCP6.0 in the report of IPCC 2014). Despite the high number of studies that have investigated how rates of calcification are affected by ocean acidification scenarios, comparatively few studies tackle how ocean acidification impacts the physiological mechanisms that drive calcification itself. Carbonic anhydrases (CAs, EC 4.2.1.1) play a major role in the physiology of coral calcification [3]. These enzymes catalyze the interconversion of CO_2_ to bicarbonate ions and protons according to the following reaction: CO_2_ + H_2_O ↔ HCO_3_^−^ + H^+^. Even if the reaction of CO_2_ hydration/HCO_3_^−^ dehydration occurs spontaneously at reasonable rates in the absence of catalysts, their presence can speed up the reaction up to 10^7^ times (hydration reaction occurs at a rate of 0.15 s^−1^ in water, whereas the rate for the most active human CA, hCAII is about 1.4 × 10^6^ s^−1^). In corals, several CAs have been identified at the molecular level in different coral species and the phylogenetic tree reveals three main clusters, the cytosolic and mitochondrial proteins, the membrane-bound or secreted proteins, and the carbonic anhydrase-related proteins [3]. These enzymes play major roles in two essential processes of coral physiology: they are involved in carbon supply for calcification as well as in carbon concentrating mechanisms for symbiont photosynthesis. However, the full molecular sequence together with the tissular localization have only been obtained for two isoforms of the coral *Stylophora pistillata* [3,4]. Both of these isoforms, STPCA and STPCA2, have been localized in the coral-calcifying cells, named calicoblastic cells. These cells also transport ions (calcium and bicarbonate) [5,6,7], regulate pH at the site of calcification [8], and synthesize organic matrix molecules which are then incorporated in the skeleton [9]. In the process of calcification two roles have been attributed to the two CA isoforms in *S. pistillata*: (1) STPCA catalyzes the interconversion between the different inorganic forms of dissolved inorganic carbon at the site of calcification [3,9,10]; (2) STPCA2 is an intracellular enzyme which is then found as an organic matrix protein incorporated in the skeleton [11,12,13]. As is the case for other enzymes, carbonic anhydrases are sensitive to environmental conditions and the pH dependency of the activity of bovine CA is well described [14,15]. Contrarily to mammals, to our knowledge, there are no data in corals concerning the dependency of the activity of carbonic anhydrase isoforms as a function of pH. The aim of our paper was, thus, to determine how the carbonic anhydrases characterized in corals are regulated by ocean acidification. For this we measured, *in vitro*, the kinetic constant (kcat) and the catalytic efficiency (kcat/Km where Km is the Michaelis-Menten constant) which both reflect the enzyme activity of STPCA and STPCA2 under a range of pH from 6 up to 9.5. In addition, we looked at gene expression of these enzymes *in vivo*, in corals maintained under conditions of CO_2_-driven seawater acidification from pH 8 down to values of pH 7.2. This range of seawater pH has proved informative in several previous investigations that sought to identify clear patterns of physiological responses in corals under seawater acidification.

## 2. Results and Discussion

### 2.1. pH Dependency of Coral Carbonic Anhydrases

**Figure 1:**
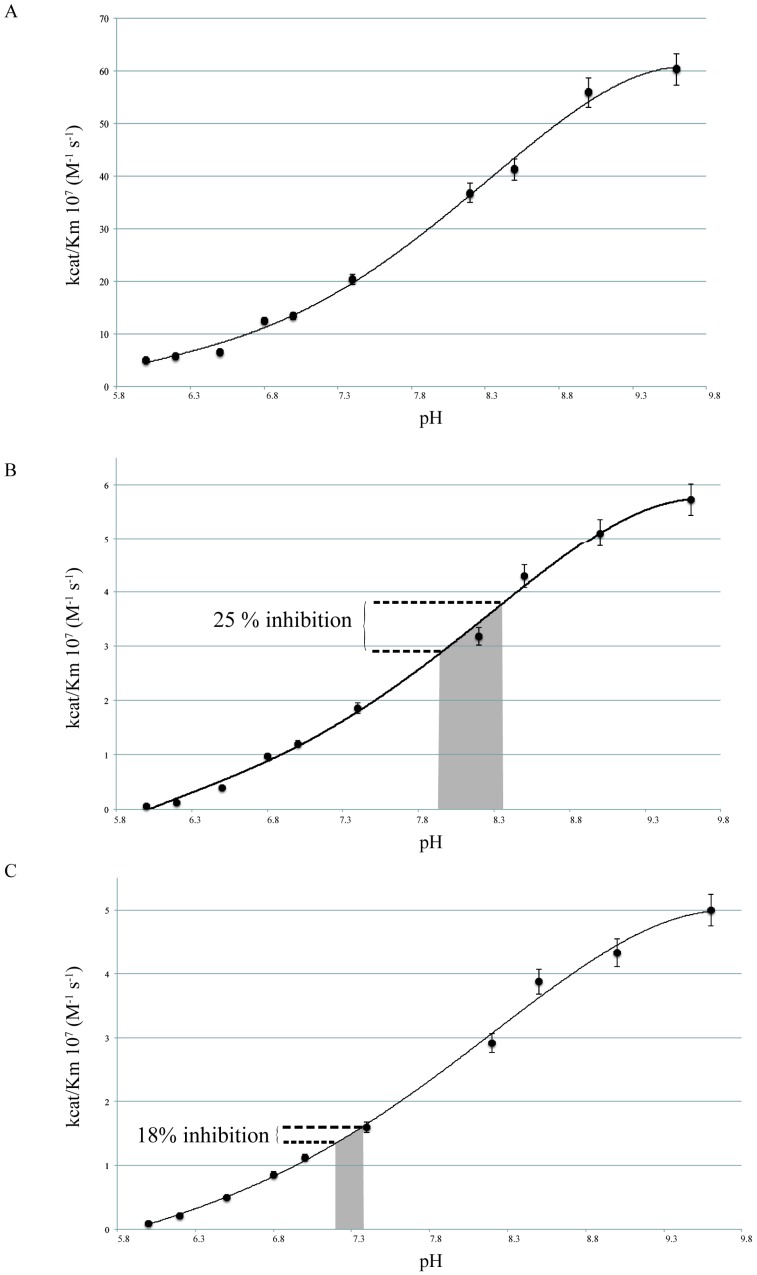
Catalytic efficiency (rate constant kcat/Km) for CO_2_ hydration of carbonic anhydrase isoforms as a function of pH for (**A**) human CA II (hCAII); (**B**) coral STPCA; and (**C**) coral STPCA2. Decrease in catalytic efficiency for the coral CAs due to an increase in acidification between pH 8.36 to 7.93 for STPCA and between pH 7.38 to 7.19 for STPCA2 is highlighted in grey.

The pH dependency of CAs is primarily due to the protonation state of Zn-bound water at the active site. The curve describing the pH dependency of mammalian CAs activity for hydration of CO_2_ is typically sigmoidal with a plateau obtained for alkaline values, a decrease in enzyme activity with decreasing pH, and a plateau for the most acidic values [14,15]. As can be seen on Figure 1, the catalytic efficiency (kcat/Km) of human CAII (hCAII) and two coral CAs, STPCA and STPCA2, shows a sigmoidal curve with a similar IC_50_ around 7.9. The linear part of the curve of enzyme activity *vs.* pH is obtained in the same range of pH for the three enzymes (between 7 and 8.9). STPCA is the membrane bound/secreted isoform localized in the calcifying cells and this enzyme is supposed to play a key role by modifying the kinetics of CO_2_/HCO_3_^−^ hydration reactions at the site of calcification [3,10,16]. The linear part of the curve for STPCA fits within the physiological range of this enzyme as pH at the site of calcification varies during diurnal cycles [17,18]. For STPCA2, which has been localized in the cytosol of calcifying cells, the linear part of the curve fits within the physiological pH value which remains almost constant at a pH of 7.4 during the diurnal cycle [18].

### 2.2. pH Dependency of Coral Carbonic Anhydrases: Effect of Ocean Acidification

**Figure 2:**
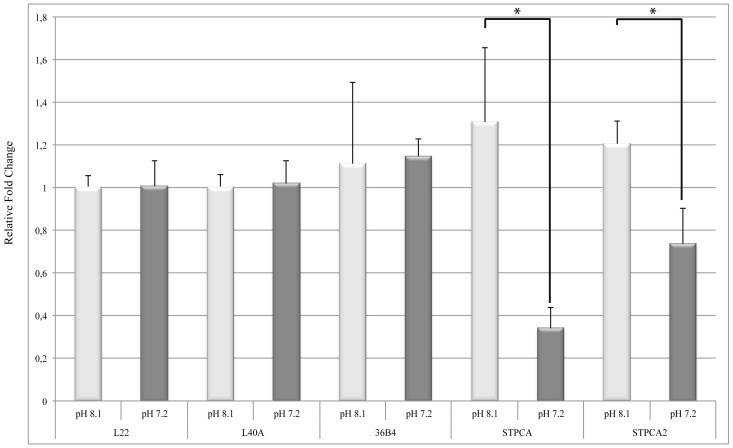
Relative gene expression of STPCA and STPCA2 by qPCR in *Stylophora pistillata*. Gene expression is relative to RPL22 expression, as well as RPL40A or RPLP0 (36B4) expression. Gene expression was measured in control sea water (pH 8.1 light grey) or after one-year exposure to a pH of 7.2 (dark grey). Errors bars represent standard error of the mean. * One-way ANOVA with *p* < 0.05.

Mechanistic studies on the response of corals to ocean acidification rely on physiological [8,17,18] and transcriptomic data [19,20,21,22,23,24]. It has been shown that the expression of several proteins changes under ocean acidification, some of them being upregulated, whereas others are downregulated. Moya *et al.* [21] have observed that in coral larvae, the expression of an *Acropora millepora* membrane/bound CA orthologous to STPCA is decreased under short-term exposure to moderate acidification (pH 7.96 and 7.86). Vidal-Dupiol *et al.* [24] observed that genes coding for CAs (with significant similarities with proteins that were previously shown to be involved in *Stylophora pistillata* calcification) were upregulated at moderate pH values of 7.8 and 7.4, but downregulated at the extreme level of pH 7.2 for the adult coral *P.* *damicornis* during a three-week exposure. Rocker *et al.* [23] showed that there was no change in genes coding for CAs for the adult coral *A. millepora* after 14 days of exposure to a pH of 7.57 (these CAs are not orthologous neither to STPCA nor to STPCA2). Hoadley *et al.* [25] have reported that there is no effect on gene expression of extra- and intra-cellular CAs (respectively, orthologous to STPCA and STPCA2) for two adult corals *P. damicornis* and *A. millepora* after 24 days of exposure to pH 7.90 and 7.83. Such discrepancies in the results have been attributed to species differences and/or stage-specific responses and/or experimental conditions. In the present study we focused on the coral *Stylophora pistillata* for which many physiological and molecular data related to calcification are available [10]. We measured the expression of genes coding for two isoforms of carbonic anhydrases, STPCA and STPCA2 (Figure 2) after one-year exposure of adult colonies to a pH of 7.2. These samples were part of a larger experiment in which we measured calcification rates and other physiological parameters linked to calcification. We have shown that calcification decreases under acidification, whereas photosynthesis and symbiont density were not affected [17]. Our present results clearly show that the effect of OA on the expression of genes coding for CAs is different when considering STPCA or STPCA2 with 3.85-fold and only 1.64-fold under-expression, respectively.

**Figure 3:**
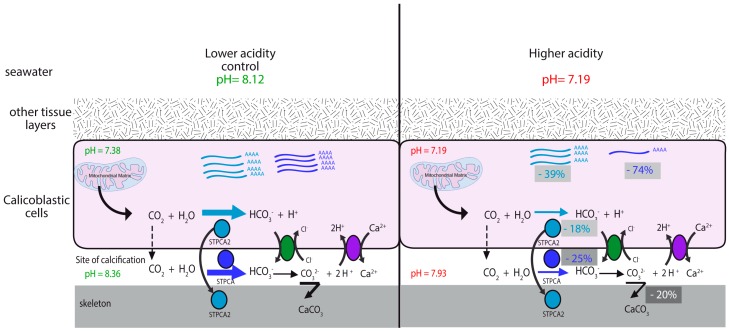
Schematic representation of the impact of ocean acidification on STPCA and STPCA2. Under seawater acidification, the intracellular pH decreases together with the pH at the site of calcification [8,16]. In the present study we have shown that under these conditions the expression of the transcripts coding for the intracellular CA isoform, STPCA2, and the membrane-bound/secreted isoform STPCA, decreases by, respectively, 39% and 74%, and their activity decreases, respectively, by 18% and 25%. This decrease of both gene expression and enzyme activity will affect the CO_2_/HCO_3_^−^ hydration and can explain that there will be less bicarbonate (and ultimately carbonate) available for the calcification process (calcification is decreased by 20% under these conditions).

The range of physiological values that enzymes face within the coral when external seawater pH decreases from 8.0 to 7.2 is different for these two enzymes. At the site of calcification, STPCA, the membrane bound/secreted isoform, faces a decrease of 0.43 pH units (from 8.36 to 7.93, [17]). Within this range of pH, the activity of STPCA (kcat/Km) decreases of 25% (Figure 1). In the cells, STPCA2 faces a change in pH of only 0.19 (from 7.38 to 7.19, [8]) while its activity (kcat/Km) decreases of 18% (Figure 1). Thus, under acidification there is, at the same time, both an under-expression of the two isoforms of CAs and an inhibition of their activity (see schematic representation Figure 3). The results that we obtained during this experiment show that calcification is affected (rates of calcification measured by the buoyant weight technique decreased by about 20% at pH 7.2 compared to pH 8) with more porous skeletons under acidification [17]. We have shown that the decrease in pH at the site of calcification and inside the cells, together with a decrease in organic matrix proteins content, can explain such a pattern [17]. The results of the present study clearly show that CAs are affected by acidification. This enzymatic response could, thus, be another parameter which explains that calcification is affected under acidification, as suggested in Venn *et al.* [8].

### 2.3. pH and Temperature Dependency of Coral Carbonic Anhydrases

**Figure 4:**
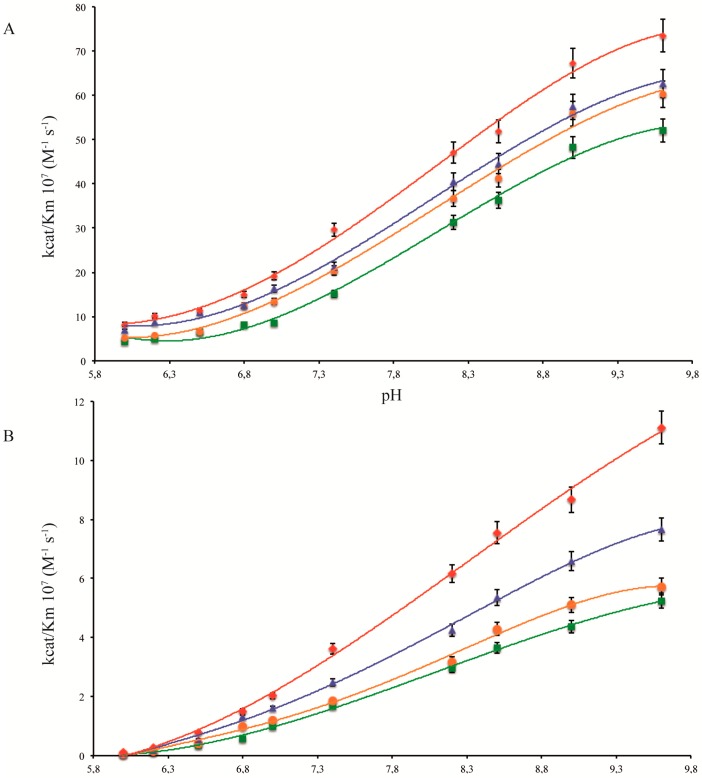

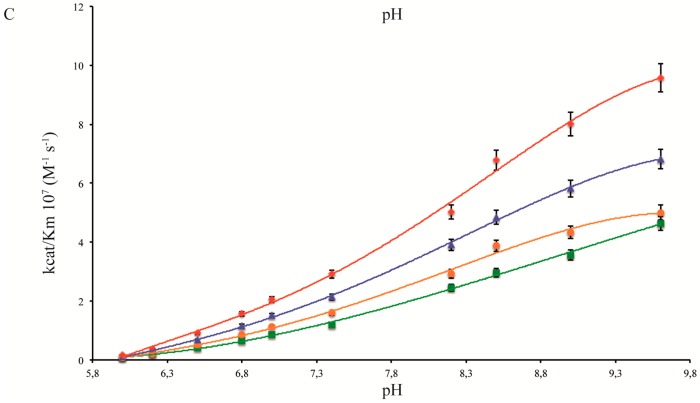
Catalytic efficiency (rate constant, kcat/Km) for CO_2_ hydration activity of carbonic anhydrase isoforms as a function of pH at different temperatures (**A**) human CA II (hCAII) (**B**) coral STPCA, and (**C**) coral STPCA2. pH variation is measured at 23 °C (■ green), 25 °C (● orange), 28 °C (▲ blue), and 31 °C (♦ red).

In this study we looked at ocean acidification, one of the side effects of the increase in atmospheric CO_2_. Another one is global warming of the oceans [26]. As for pH, different scenarios of temperature increase have been proposed (IPCC, 2014) [1], depending on greenhouse gas emissions, with RCP2.6 being representative of a scenario that aims to keep global warming likely below 2 °C above pre-industrial temperatures. Since in the future ocean corals will face the combined effect of temperature increase and pH decrease, we have, thus, looked at the activity of STPCA and STPCA2 when these two stressors are combined. As can be seen on Figure 4, for a given pH, CA activity (kcat/Km) increases with increasing temperature which is usually observed for enzymes when they work in their physiological temperature range. However, what is noteworthy is that for a combined increase in temperature and decrease in pH, there is an opposite effect on CA activity (kcat/Km) suggesting that the effect of one of these stressors can counterbalance the effect of the other. For example, the catalytic constant (kcat) of STPCA at the site of calcification is similar at control pH and control temperature (25 °C and pH 8.36) as at increased acidification and increased temperature (28 °C and pH 7.93, Table 1) since the decrease in CA activity when pH decreases is counterbalanced by the increase in CA activity when temperature increases. The same effect is observed for STPCA2 where the catalytic constant is even slightly higher under acidification, combined with increased temperature than in control conditions (Table 1). There are only four studies that have looked at the combined effect of temperature increase and pH decrease on gene expression of CAs. Two carbonic anhydrase transcripts were down regulated in the coral *A. aspera* after a 14 day exposure at pH 7.9 and 35.2 °C (compared to control at pH 8.1 and 31 °C; [22]), two CAs transcripts were upregulated in the coral *A. millepora* after a 21 day exposure at pH 7.98 and 30.83 °C compared to control at pH 8.15 and 28.07 °C [23], six CA transcripts were downregulated in *A. millepora* after a five week exposure to pH of 7.85 and 7.68, with respective temperatures of 26 °C and 28 °C compared to control conditions at pH 8.02 and 24 °C [20]. Finally, another study, on *A. millepora* and *P. damicornis* CAs orthologous to *S. pistillata* STPCA and STPCA2, was performed during a 24-day exposure to pH 7.83, 7.9, and 8.07 (control) at two different temperatures (control 26.5 °C and 31.5°). It was observed that gene expression was only affected for the intracellular isoform of *A. millepora* under a temperature increase [25]. The different trends in gene expression in these four studies can be explained, for example, by a difference in the experimental protocols (different pH/temperature values, different time of exposure), or by a difference in the CA isoforms that were measured (however, molecular data on CAs are not available for all these studies). Regardless of the trend in gene expression, our results show that changes in CA activity with increasing temperature/decreasing pH can modulate the effect of the stressors on gene expression. Studies dealing only with the effect of temperature show that CA gene expression is downregulated when temperature increases [27,28,29,30], but in light of our results, we suggest that this could be, at least in part, counterbalanced by an increase in enzyme activity. However, it is not possible to determine quantitatively how respectively gene expression and enzymatic activity affect the physiological function of the enzyme.

## 3. Material and Methods

*Biological material and treatments—*Colonies of the tropical coral *Stylophora pistillata* were exposed to one-year seawater acidification as described previously [8,17]. Briefly corals were kept in aquaria supplied with Mediterranean seawater (exchange rate 70%/h) at a salinity of 38, temperature 25 C and irradiance of 170 μmol photons m^−2^∙s^−1^ on a 12 h/12 h photoperiod provided by HQI-10,000K metal halide lamps (BLV Nepturion, Steinhöring, Germany). Carbonate chemistry was manipulated by bubbling with CO_2_ to reduce pH to the target values of pH 7.2. Control treatment was pH 8.1. Values of carbonate chemistry parameters are those measured in Tambutté *et al.* [17].

*CA activity—*An Applied Photophysics stopped-flow instrument has been used for assaying the CA-catalyzed CO_2_ hydration activity [14]. Assay was performed on recombinant human and coral CAs (hCAII, STPCA, STPCA2, [4,31,32,33]). Phenol red (at a concentration of 0.2 mM) was used as indicator, working at the maximum absorbance of 557 nm, with 10 mM TRIS at ten different pH levels (6.0; 6.2; 6.5; 6.8; 7.0; 7.4; 8.2; 8.5; 9.0; 9.6), and 20 mM Na_2_SO_4_ or 20 mM NaCl (for maintaining constant the ionic strength), following the CA-catalyzed CO_2_ hydration reaction for a period of 10–100 s. The CO_2_ concentrations ranged from 1.7 to 17 mM for the determination of the kinetic parameters and inhibition constants. For each inhibitor at least six traces of the initial 5%–10% of the reaction have been used for determining the initial velocity. The uncatalyzed rates were determined in the same manner and subtracted from the total observed rates. Stock solutions of inhibitor (1 mM) were prepared in distilled-deionized water with 10%–20% (*v*/*v*) DMSO (which is not inhibitory at these concentrations) and dilutions up to 0.01 nM were done thereafter with distilled-deionized water. Inhibitor and enzyme solutions were preincubated together for 15 min at room temperature prior to assay, in order to allow for the formation of the E–I complex. The inhibition constants were obtained by non-linear least-squares methods using PRISM 3, from Lineweaver-Burk plots, as reported earlier, and represent the mean from at least three different determinations.

The temperature was controlled by an automatic thermostat, with a precision of ±0.2 °C. The solution of substrate and enzyme were thermostated at the required temperatures for 30 min before assay, and the same temperatures have been applied to the spectrophotometric cell where the reaction occurred.

*Real-Time PCR experiments*—Total RNAs extraction and cDNA synthesis were performed as described previously [34]. Briefly, cDNAs were synthesized using the Superscript^®^III kit (Invitrogen, Courtaboeuf, France). The experiment was repeated three times on clonal individuals. For each biological replicate, real-time PCR was then performed in technical triplicate with cDNAs diluted at a final concentration of 2 ng/μL and using the Express SYBR^®^ greenER™ SuperMix with premixed ROX (Invitrogen, Courtaboeuf, France) in ABI 7300 Real-Time PCR System (Applied Biosystems, Courtaboeuf, France). Primers used (STPCA, STPCA2,) are from [35] and control gene 36B4 from [34]. We used two other control genes, ribosomal protein L22 (L22 Forward: 5′-TGATGTGTCCATTGATCGTC-3′ and L22 Reverse 5′-CATAGGTAGCTTGTGCAGATG-3′) and L40A genes (L40A Forward: 5′-CGACTGAGG GGAGGAGCCAA-3′ and L40A Reverse 5′-CTCATTTGGACACTCCCTT-3′). Relative expressions were calculated using Biogazelle qbase + 2.6™ (Gent, Belgium). Results are presented as mean ± SEM. Data were checked for normality using a Kolmogorov–Smirnov test with Lilliefors correction and log-transformed, if required. One-way ANOVA was used to test the effect of pH on STPCA and STPCA2. Differences were considered significant for *p*-values < 0.05. Statistics were performed using Statistica 10 (Statsoft, Tulsa, OK, USA).

## 4. Conclusions

Our results on the response of carbonic anhydrases to ocean acidification in the coral *Stylophora pistillata* show that these enzymes are affected by ocean acidification via an effect on both gene expression and enzyme activity. Our results also clearly show that temperature increase affects CA activity and we suggest that this could counterbalance the effect of acidification. Finally, we point out that caution must, thus, be taken when interpreting transcriptomic data on CAs in ocean acidification and temperature stress experiments as the effect of these stressors on the physiological function of CAs will depend both on gene expression and enzyme activity.

## Figures and Tables

**Table 1:** Catalytic activity (kcat) of coral carbonic anhydrase isoforms at different temperatures and pH. The values of kcat for a decrease in pH observed at the site of calcification and inside the calcifying cells (when seawater pH is decreased from control to 7.19) is highlighted in green boxes for STPCA and in red boxes for STPCA2.

	STPCA		STPCA2	
pH	25 °C	28 °C	25 °C	28 °C
**8.36**	3.943 × 10^6^ s^−1^	4.929 × 10^6^ s^−1^	3.200 × 10^6^ s^−1^	4.309 × 10^6^ s^−1^
**7.93**	2.965 × 10^6^ s^−1^	3.766 × 10^6^ s^−1^	2.410 × 10^6^ s^−1^1	3.286 × 10^6^ s^−1^
**7.38**	1.856 × 10^6^ s^−1^	2.401 × 10^6^ s^−1^	1.494 × 10^6^ s^−1^	2.098 × 10^6^ s^−1^
**7.19**	1.530 × 10^6^ s^−1^	1.981 × 10^6^ s^−1^	1.221 × 10^6^ s^−1^	1.737 × 10^6^ s^−1^

## Abbreviations

CA	Carbonic Anhydrase
STPCA	*Stylophora pistillata* carbonic anhydrase (ACA53457)
STPCA2	*Stylophora pistillata* carbonic anhydrase (ACE95141)
OA	Ocean Acidification
